# Quantification of cervical spine muscle fat: a comparison between T1-weighted and multi-echo gradient echo imaging using a variable projection algorithm (VARPRO)

**DOI:** 10.1186/1471-2342-13-30

**Published:** 2013-09-11

**Authors:** James M Elliott, David M Walton, Alfred Rademaker, Todd B Parrish

**Affiliations:** 1 Department of Physical Therapy and Human Movement Sciences, Feinberg School of Medicine, Northwestern University, 645 North Michigan Avenue, Suite 1100 room 1139, Chicago, IL 60611, USA; 2 School of Physical Therapy, Western University, London, Ontario, Canada; 3 Department of Preventive Medicine, Feinberg School of Medicine, Northwestern University, Chicago, IL USA; 4 Department of Biomedical Engineering, McCormick School of Engineering, Northwestern University, Evanston, IL USA; 5 Department of Radiology, Feinberg School of Medicine, Northwestern University, Chicago, IL USA

## Abstract

**Background:**

Previous data using T1-weighted MRI demonstrated neck muscle fat infiltration (MFI) in patients with poor functional recovery following whiplash. Such findings do not occur in those with milder symptoms of whiplash, chronic non-traumatic neck pain or healthy controls, suggesting traumatic factors play a role. Muscle degeneration could potentially represent a quantifiable marker of poor recovery, but the temporal constraints of running a T1-weighted sequence and performing the subsequent analysis for muscle fat may be a barrier for clinical translation. The purpose of this preliminary study was to evaluate, quantify and compare MFI for the cervical multifidus muscles with T1-weighted imaging and a more rapid quantitative 3D multi-echo gradient echo (GRE) Dixon based method in healthy subjects.

**Methods:**

5 asymptomatic participants with no history of neck pain underwent cervical spine MRI with a Siemens 3 Tesla system. The muscle and fat signal intensities on axial spin-echo T1-weighted images were quantitatively classified for the cervical multifidii from C3-C7, bilaterally. Additional axial GRE Dixon based data for fat and water quantification were used for comparison via paired t-tests. Inter-tester reliability for fat and water measures with GRE images were examined using 1) Pearson’s Intra-class correlation coefficient 2) Bland-Altman Plots and 3) Lin’s-Concordance Coefficient. P < 0.05 was used to indicate significance.

**Results:**

Total mean (SD) MFI (C3-C7) for the multifidii obtained with T1-weighted imaging and GRE were 18.4% (3.3) (range 14-22%) and 18.8% (2.9) (range 15-22%), respectively. The Pearson correlation coefficients for inter-tester reliability on the GRE sequences for the C3-C7 multifidii ranged from .83 - .99, indicating high levels of agreement with segmental MFI measures. Bland-Altman Plots revealed all data points were within 2 SDs and concordance was established between 2-blinded raters, suggesting good agreement between two raters measuring fat and water with GRE imaging.

**Conclusions:**

Results of this preliminary study demonstrate reliability between 2 raters of varying experience for MRI analysis of MFI with 3D GRE MRI. The quantification of MFI for healthy cervical musculature is comparable to T1-weighted images. Inclusion of larger samples of symptomatic data and histological comparison with the reference standard biopsy is warranted.

## Background

Magnetic resonance imaging (MRI) has been used to quantify muscular fatty infiltrates (MFI) in patients with acute and chronic neck disorders [1-4], low back pain [5-8] and a number of neuromusculoskeletal conditions [9-11]. Cross-sectional investigations have determined significantly lower magnitudes of neck MFI in those with non-traumatic, insidious onset pain when compared to those with chronic whiplash related pain [3], suggesting traumatic factors play a role in their development. Using conventional T1-weighted MRI in prospective fashion, MFI were shown to develop between 4-weeks and 3-months post whiplash injury in patients with moderate to severe levels of pain and disability but not in those with lower levels of acute pain [2]. Findings of muscle degeneration in those with higher levels of pain and disability following whiplash injury from a motor vehicle crash could potentially represent a quantifiable marker of poor recovery. As such, the early radiological observation and quantification of MFI may prove informative for clinical practice. However, a potential challenge in translating such findings remains the temporal constraints of running the longer T1-weighted sequence and performing a quantitative analysis for MFI especially when considering patient throughput in a busy radiology practice.

There are however a number of other more rapid methods for quantifying the water and fat composition of a voxel when compared to T1-weighted imaging. These include a dual acquisition method, where the water frequency is selectively excited (water image) [12] and a standard image is collected (fat and water image). This however relies on the uniform frequency difference between water and fat across the whole volume of excitation, which is often difficult to obtain at higher magnetic fields. A fat suppressed acquisition using a short-tau inversion recovery (STIR) sequence is possible, but the T1 of fat has to be assumed, which may vary depending on the evolution of the infiltration of the muscle [13]. An alternative is the Dixon method [14], where one collects data at an echo time when water and fat are in-phase and at an echo time when the phases are opposed. The data can be combined to generate a fat and water image but is prone to errors in the presence of field inhomogeneities. Collecting multiple echo time data can improve the estimation of the fat and water images and this technique has been applied successfully in the musculoskeletal system [15], adrenal glands [16], kidneys [17] and liver [18,19]. The measure has recently expanded to include the quantification of intramuscular fat of the lower extremity in patients with myopathy [9] and in the lumbar paraspinals of a large population of patients with low back pain [20]. The proposition solicits that such rapid 3D imaging methods could be used to quantify fat content of the neck muscles. It is not possible however to offer physiological interpretation of such data without producing normative reliability data as a foundation.

The intent of this preliminary study was to 1) compare the measurement of cervical muscle fat using 3D Gradient Echo (GRE) multi-echo fat/water acquisitions to a previous measure using T1-weighted sequences and 2) establish inter-rater reliability for measuring cervical muscle fat and water using 3D fat/water separation MRI. We chose the GRE sequence because of the more time efficient data collection of the GRE sequence. We expect that the fat and water measure will provide valid quantification of muscle fat compared to that of the previously established and reliable T1-weighted measure [1], while offering the important clinical advantages of ease and efficiency.

## Methods

### Study population

3 healthy male and 2 female volunteers (mean age (SD) [range]- 35 (8.6) [26–48]), with no previous history of neck pain or trauma to the cervical spine, were sought for the study. Only males and females aged 18–55 years were considered in this first instance. There were several criteria that excluded potential participants, which related to their neuromusculoskeletal status and institutional guidelines for the safe application of MRI to asymptomatic volunteers. They included: a previous history of neck pain, vertiginous symptoms, a history of a motor vehicle accident, neurological conditions, inflammatory joint disease or systemic disease e.g. diabetes. In relation to the MRI scan, volunteers were not considered if they suffered claustrophobia, had a pacemaker, aneurysm clip or any small implanted metal hardware or wires that could move or be affected when introduced to a magnetic field. Furthermore, subjects were not considered if they were pregnant or if, in the absence of an effective form of contraception, they could possibly have conceived since the first day of their last normal menstrual period.

Healthy subjects were recruited from the university community in urban Chicago, Illinois, USA. Eight volunteers responded to the appeals for participants. In total, 3 out of 8 potential participants were excluded secondary to a previous motor vehicle accident (1), claustrophobia (1) and pregnancy (1). Thus, 5 healthy subjects were included (2 males and 3 females).

Ethical clearance was obtained from the Northwestern University Institutional Review Board, Chicago, Illinois, USA. All subjects provided written informed consent to participate. All data were stored in a secured database and de-identified.

### Measurements

#### Demographic and anthropometric data

Subjects completed a baseline questionnaire that included details on their age, height, weight, and an institutional checklist of absolute and relative contraindications for MRI.

#### MRI examinations

All examinations were performed with a 3.0 T MRI scanner (Trio, Siemens, Erlangen, Germany). Each participant underwent MR examination of the cervical spine (from the cephalad portion of C2 to the caudal portion of C7). In addition to the standard 12-channel head coil, a dedicated neck coil of the manufacturer was used as a receiver coil. Once positioned in the magnet, a localizer scan and a T2-weighted sagittal turbo spin echo sequence were performed.

#### T1-Weighted MRI

The T1-weighted axial scan of the cervical spine was planned from the T2-weighted sagittal sequence and the slab was manually angled perpendicular to the normal lordotic curve of each subject using the middle of the C2/3 intervertebral disc as a reference point. The slab was then manually positioned from the cephalad portion of C2 through the caudal portion of the C7 vertebral end plate to ensure proper capture of the cervical extensor musculature. Parameters consisted of: slices = 24, slice thickness of 4 mm and a gap of 0.8 mm, FOV = 200 × 200 mm, TR = 448 ms, TE = 14 ms, matrix size = 256*256, in plane resolution of 0.9 mm × 0.9 mm, acquisition time TA = 8:42 seconds.

This established measure of relative fat within the muscle [1,3,4,21] was used to quantify a pixel intensity profile for both muscle and fat with AnalyzeDirect software (V. 11.0). The measure, based on the contrasting signal intensities of fat and water on T1-weighted imaging, allows for the simple process of quantifying relative amounts of intramuscular fat compared to a recognized area of intermuscular fat at the C2 level [21]. Analysis consisted of manually tracing user-defined regions of interest (ROI) over the bilateral multifidii muscles on the axial T1-weighted images at the most cephalad portion of the vertebral bodies of C3 – C7.

Histograms were created from the summated user-defined ROI, displaying each particular image pixel intensity profile. A numerical representation of each image muscle and intermuscular fat pixel intensity profiles were created and the text file was exported to an Excel ® spreadsheet for calculation of the MFI for T1-weighted imaging. Dividing the mean pixel intensity of the muscle ROI by the mean pixel intensity of the intermuscular fat ROI created the MFI value.

#### 3D Multi-echo Dixon fat/water imaging

A number of imaging techniques, based on the different precessional frequencies of fat and water protons can be used to quantify fat and water concentration. We used a three-dimensional multi-echo gradient echo acquisition to collect the data required for the analysis of phase related to the precessional differences in fat and water. The Fast Low Angle Shot (FLASH) based axial gradient echo sequence parameters were TR = 23.81 ms, 8 echo times with a spacing of 1.78 ms starting at 1.36 ms. A single slab was placed over the cervical spine with 36 partitions and a partition thickness of 3 mm and slab oversampling of 22% to prevent aliasing in the 3D direction. The in-plane resolution was 1.4 mm using a rectangular field of view of 75% resulting in an acquisition time of 2:06 minutes. This scan also covered the cephalad portion of C2 through the caudal portion of the C7 vertebral end plate.

#### Fat/water quantification

The water separation algorithm uses the variable projection (VARPRO) method to find an iterative solution for the entire image [22]. The VARPRO algorithm can use four or more echoes with non-uniformly spaced echo times (phase shifts between water and fat) to solve the nonlinear problem with a globally optimized solution. The reconstruction process was automated in the Siemens sequence environment so that fat-only, water-only, and calculated in-phase and opposed-phase images were created. The MFI from 3D fat/water separation imaging was created from the mean pixel intensity of fat-only (Fat) and the mean pixel intensity of water-only (Water) images with the following equation:

$$\mathrm{Fat}/\left(\mathrm{Fat}+\mathrm{Water}\right)*100$$

#### Procedure

Subjects were requested to lie supine inside the magnet with a foam pad under their knees and foam padding placed on top of the head as well as on the right and left of the head to position the cervical spine in the neutral position and reduce any head movement during the exam. They were asked to lie completely still during the examination with communication between subject and operator occurring between each sequence to ensure participant comfort.

Two raters, one with 10 years of experience measuring muscle structure and one with 3-months, were tasked with establishing repeatability of MFI measures in the bilateral cervical multifidus muscle at C3 through C7 with the fat/water images. Regions of interest were drawn using AnalyzeDirect Software (V. 11.0) of the bilateral multifidii muscles at the most cephalad portion of each respective vertebral level (C3-C7). The right and left sides were combined for the analysis. The rater with less experience (rater 2) was trained by the investigator with more experience (rater 1). Each rater measured the MFI independently and this data was used for analysis. Repeatability of the T1-weighted measure has been established previously [1,21].

#### Statistical analysis

Data analysis was performed with the procedures implemented by using SPSS statistical software (Version 20.0 for Mac; Chicago, IL). Participant demographic data are presented as proportions or means ± SD (range). The inter-rater reliability for the 3D fat/water measurements of MFI was tested using the Intraclass Correlational Coefficient (ICC_3,1_ - *which is used when all subjects are rated by the same raters who are assumed to be the entire population of raters*), Bland & Altman plots and Lin’s Concordance Coefficients to assess and confirm levels of consistency and agreement of MFI. Because the calculated MFI’s on both sequences were found to be normally distributed using the Kolmogorov-Smirnov method, the differences in MFI between the measures were evaluated by a parametric paired *t*-test with a level of significance set at α = 0.05. The inter-rater reliability for the T1-weighted measure of MFI was tested using the ICC_3,1_.

#### Sample size

Walter and colleagues have presented a robust mathematical algorithm for predicting adequate sample size for reliability studies [23]. Assuming a minimum agreement (ICC) of 0.80, with a null hypothesis (level below which we would deem the tool not reliable) of 0.4, a minimum of 9 independent observations provides an acceptable 5% alpha error rate (p < 0.05) and 20% beta error rate. In order to produce a more robust estimate, right and left multifidus was measured from C3-C7 by each rater. As such, each image represented 10 data points (5 pairs of data). The images were presented in a randomized fashion and thus, error was also random.

## Results

**Figure 1:**
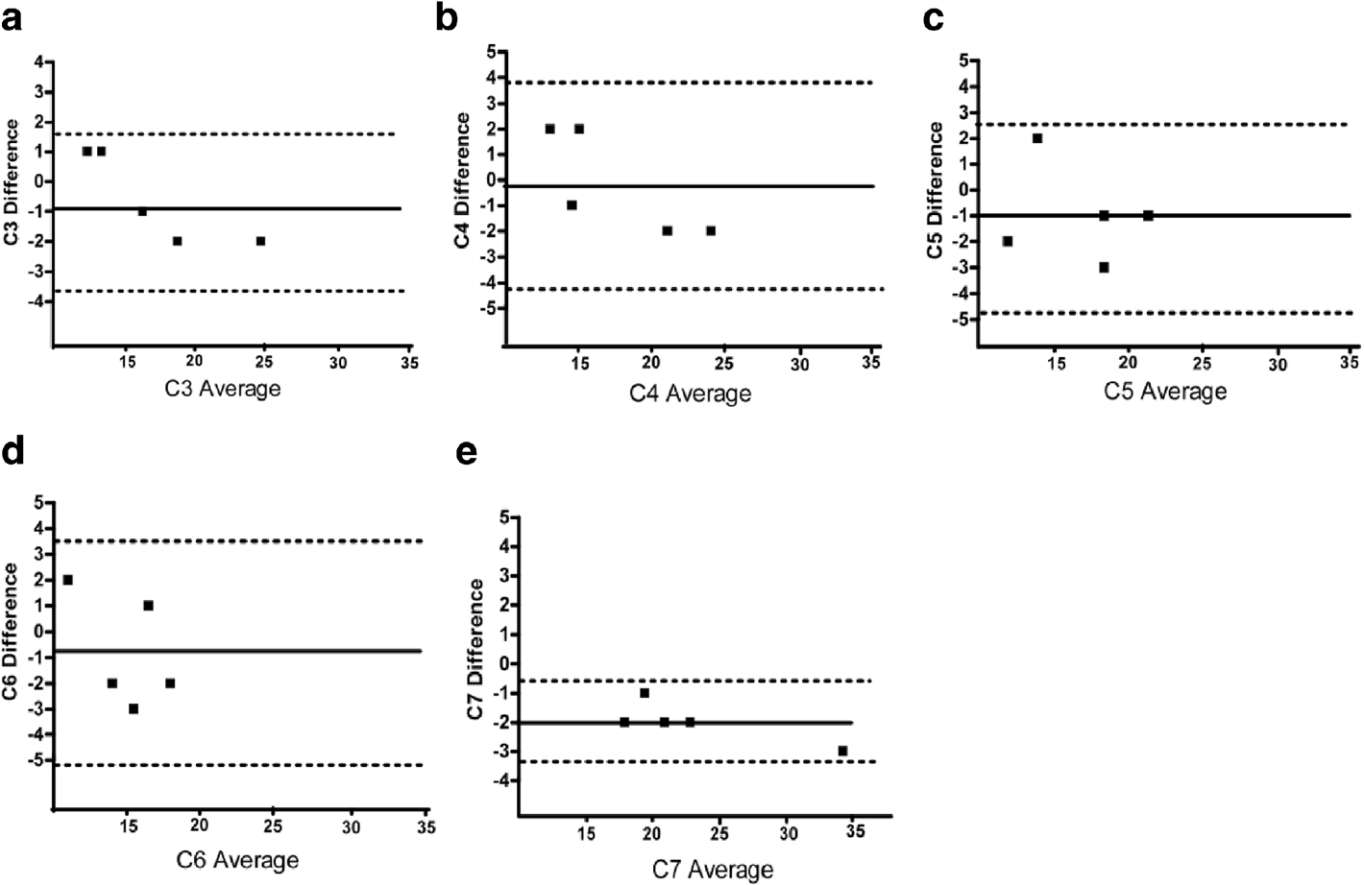
**Bland-Altman Plots of C3-C7 multifidus MFI difference vs. average between 2-raters: a) C3, b) C4, c) C5, d) C6 and e) C7.** Data was acquired from the 3D multi-echo GRE fat/water separation sequence.

**Figure 2:**
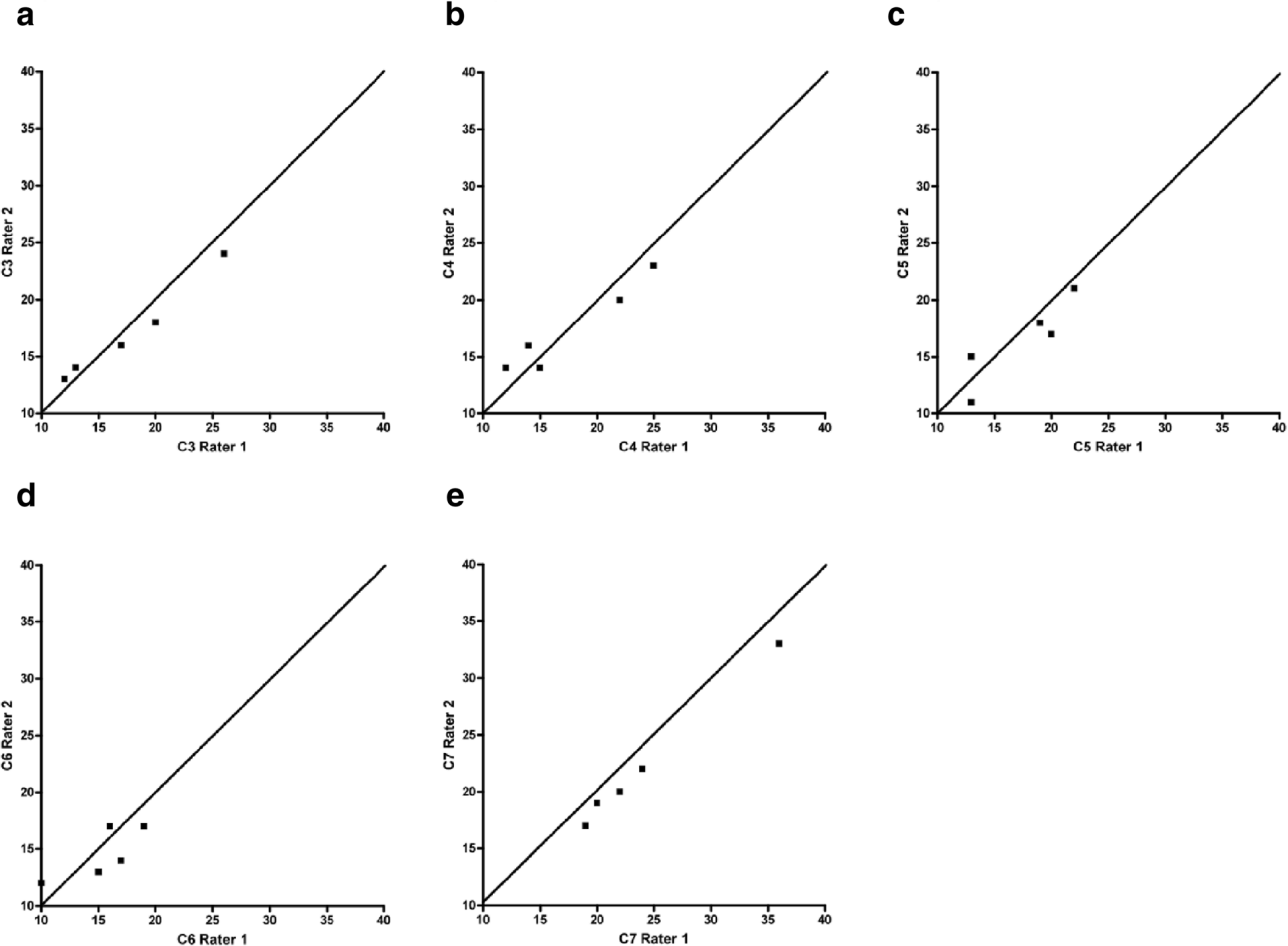
**Lin Concordance Coefficients for segmental multifidus MFI between 2-raters: a) C3, b) C4, c) C5 d) C6 and e) C7.** Data was acquired from the 3D multi-echo GRE fat/water separation sequence.

The subjects’ characteristics in relation to age and BMI are presented in Table 1. The ICCs for the inter-rater reliability using the fat/water sequence of the multifidus ranged from .83 - .99 indicating good to excellent inter-rater reliability and consistent with previously established data (Table 2) [1,21]. Figure 1a-e details the results from Bland & Altman Plots for the fat/water imaging measure, which reveals that both rater differences for MFI were within +/− 2 standard deviations and Figure 2a-e details Lin Concordance Coefficients, confirming concordance between 2 raters for segmental MFI of the cervical multifidus as measured by fat/water imaging. The values from Lin Concordance Coefficients are presented in Table 3.

**Table 1:** The age and BMI (Means ±SD [range]) of the asymptomatic cohort (n = 5)

	**Age (years)**	**BMI (lbs/in**^**2**^**)**
Total population (n = 5)	35.0 ± 8.6	27.4 ± 2.4
	[26 – 48]	[24.4 – 30.5]

**Table 2:** The average measures intra-class correlation coefficients [95% CI] of the inter-observer reliability of cervical multifidii MFI on 3D Multi-Echo GRE Imaging

	**Rater 1 vs. Rater 2 ICC [95% CI]**	**p - value**
C3	.97 [.78 - .99]	0.001
C4	.95 [.55 - .99]	0.006
C5	.94 [.43 - .99]	0.009
C6	.83 [.60 - .98]	0.05
C7	.99 [.97 - .99]	0.001

**Table 3:** Means for rater 1 and rater 2 (+/− SD) from Lin Concordance Coefficients for MFI of the cervical multifidus with 3D Multi-Echo GRE Imaging

	**Mean (SD) 3D GRE - MFI**		**concordance**	**p – value**	**95% CI**
	**Rater 1**	**Rater 2**			
C3	17.6 (5.6)	17 (4.4)	.95	< 0.001	.82 - .98
C4	17.6 (5.6)	17.4 (3.9)	.90	< 0.001	.69 - .98
C5	17.4 (4.2)	16.4 (3.7)	.86	0.018	.22 - .98
C6	15.4 (3.4)	14.6 (2.3)	.69	0.08	−0.10 - .94
C7	17.6 (5.6)	17 (4.4)	.94	< 0.001	.82 - .98

The MFI values (mean (SD) [Range]) within the cervical multifidus with T1-weighted and fat/water separation MRI are presented in Table 4*.* There were no significant differences between the MRI findings of segmental (C3, C4, C5 and C6) or total MFI (C3-C7 combined) within the multifidus muscle with both sequences, demonstrating little discrepancy between the two measures (p = 0.47). There was however a significant difference in MFI at the C7 level where the fat/water measure was significantly higher when compared to the T1-weighted measure (p = 0.04).

**Table 4:** Mean MFI (C3-C7) from T1-weighted and 3D Multi-Echo GRE Imaging ((SD) [Range])

	**Mean T1-weighted MFP (SD) [Range %]**	**Mean 3D GRE MFP (SD) [Range %]**	**p - value**
C3	19.6% (2.4) [17 - 23]	17.6% (5.7) [12 - 26]	0.25
C4	19.2% (3.9) [13 - 24]	17.6% (5.6) [12 - 25]	0.44
C5	19.8% (2.4) [18 - 24]	17.4% (4.2) [13 - 22]	0.15
C6	17.4% (1.8) [15 - 19]	15.4% (3.4) [10 - 19]	0.09
C7	17.8% (7.4) [12 - 30]	24.2% (6.8) [19 - 36]	0.04*
TOTAL	18.8% (2.9) [15 - 22]	18.4% (3.3) [14 - 22]	0.47

* Indicates significance at p < 0.05 (two-tailed).

## Discussion

**Figure 3:**
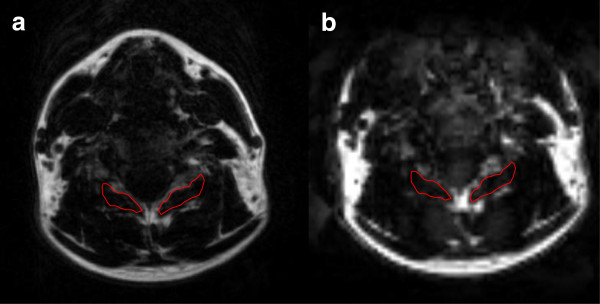
**Regions of interest measures of right and left multifidus muscle at the C5 segmental level; a)** T1-weighted magnetic resonance image **b)** fat-only image derived from the 3D VARPRO acquisition. Images were co-registered.

The results of this preliminary study involving a small sample (n = 5) asymptomatic participants indicates that the rapid acquisition 3D fat/water separation technique is comparable to T1-weighted imaging in detecting the relative fat content in the healthy cervical multifidii musculature (Figure 3). In addition, the inter-rater reliability was good to excellent for the fat/water technique, suggesting this method is reliable between a finite set of 2-raters of varying experience (ICC_3,1_) but it needs to be determined if it would be readily and rapidly replicated by different examiners (or raters) outside our laboratory (ICC_2,1_). As can be seen in Tables 2 and 3 and Figures 1 and 2, the measure of MFI with fat/water separation is repeatable between two blinded raters with varying levels of experience. Altogether, the more experienced rater 1 consistently measured higher levels of MFI when compared to rater 2. The clinical significance of this difference between raters is however unknown at this time. While experience could factor into a more liberal inclusion of musculofascial borders for the cervical multifidus on axial images, larger numbers of raters with varying levels of experience would be required before deriving definitive conclusions on the influence of experience. Furthermore, precise reasons into the significantly higher MFI at the C7 level with the 3D fat/water measure when compared to the T1-weighted measure is unknown at this time but could result from field inhomogeneities or RF profile induced signal changes at the outer edge of the excitation volume.

There is support for establishing equivalence between different methods on the basis of their ability to quantify measured samples, all of which however carry some limitations. Lin first proposed the Concordance Coefficient in 1989 for assessment of concordance in continuous data as an alternative way of assessing agreement between alternative methods, such as the Pearson correlation coefficient *r*, paired *t*-tests, coefficient of variation, and the ICC [24]. In addition, the Lin concordance coefficient has shown to be robust for small samples of data [24], such as reported in this preliminary study. Accordingly, we chose to include the Lin Concordance Coefficient in tandem with ICCs_3,1_ and Bland Altman Plots to ensure a robust statistical analysis of our repeatability when producing multiple measures for MFI in the cervical multifidus in a small healthy sample of five subjects.

Considerable evidence exists to support a relationship between muscle fatty infiltrates and painful symptoms in patients with neck [1,2,4,25-27] and low back pain as well as a host of other neuromususculoskeletal disorders [7-11,20,28,29]. A recent longitudinal study of whiplash provides preliminary evidence that the manifestation of fatty degeneration, between 4-weeks and 3-months post injury, is associated with higher levels of initial pain and signs of post-traumatic stress [2]; a robust predictor of poor-functional recovery [30,31]. Such muscle changes were not expressed in those with lower levels of initial pain suggesting their presence is unique to those who transition to chronic pain following whiplash [2] and that the observation of their development could be important in clinical MR imaging studies. However, the routine quantification of such muscle changes following whiplash is unlikely when one considers the time-constraints of currently available clinical measures. The use of rapid acquisition 3D multi-echo fat/water imaging provides a potential clinical alternative and results of this small study provide foundation for its use in quantifying the temporal degeneration of soft-aqueous neck muscles following whiplash injury in prospective fashion. Positive findings would help to translate the use of such imaging sequences to the clinical setting whereby the exploration and development of more informed (and early) management strategies could be explored and realized.

The results of this preliminary study were obtained in a small sample of asymptomatic subjects. Larger sampled studies involving symptomatic subjects are required before the findings can be generalized to patient populations and this is well underway in our laboratory. Furthermore, repeated measures experiments are required to determine repeatability error with the MRI measures over time.

## Conclusions

3D multi-echo fat/water MR imaging provides for an accurate and rapid measure of MFI for the cervical multifidus in a small sample of healthy subjects. We found excellent agreement in a small sample of healthy subjects between two-raters across multiple analysis strategies supporting the use of this method in longitudinal evaluation of temporal changes in muscle tissue structure, particularly following traumatic whiplash injury. An advantage of the 3D acquisition method is that a further time reduction could be obtained if the coverage of interest was reduced to a single level, which is not true for spin echo based imaging methods. For the future, inclusion of other muscles beyond the multifidii is required to compare MFI within and between different muscular regions. Finally, comparison of MFI with 3D multi-echo fat/water separation MR to the reference standard, muscle biopsy, is also required and this validation is underway.

## Abbreviations

MFI: Muscle fatty infiltrates; STIR: Short tau inversion recovery; GRE: Gradient echo; FLASH: Fast low angle shot magnetic resonance imaging; VARPRO: Variable projection algorithm; ICC- Intra: Class correlation coefficient; MFI: Muscle fat percentage.

## Competing interests

The authors declare that they have no competing interests.

## Authors’ contributions

Guarantor of integrity of the entire study- JE, TP, DW. Study concepts and design- JE, TP, DW. Literature research- JE, TP, DW. Clinical studies- JE, TP. Experimental studies/data analysis – JE, FR, TP. Statistical analysis- JE, FR, DW. Manuscript preparation- JE, FR, TP, DW. Manuscript editing- JE, FR, TP, DW. All authors read and approved the final manuscript.

## Pre-publication history

The pre-publication history for this paper can be accessed here:

http://www.biomedcentral.com/1471-2342/13/30/prepub
